# Skeletal Muscle myomiR Are Differentially Expressed by Endurance Exercise Mode and Combined Essential Amino Acid and Carbohydrate Supplementation

**DOI:** 10.3389/fphys.2017.00182

**Published:** 2017-03-23

**Authors:** Lee M. Margolis, Holly L. McClung, Nancy E. Murphy, Christopher T. Carrigan, Stefan M. Pasiakos

**Affiliations:** ^1^Military Nutrition Division, US Army Research Institute of Environmental MedicineNatick, MA, USA; ^2^Oak Ridge Institute for Science and EducationOak Ridge, TN, USA

**Keywords:** myomiR, load carriage, cycle ergometry, Akt, rpS6

## Abstract

Skeletal muscle microRNAs (myomiR) expression is modulated by exercise, however, the influence of endurance exercise mode, combined with essential amino acid and carbohydrate (EAA+CHO) supplementation are not well defined. This study determined the effects of weighted versus non-weighted endurance exercise, with or without EAA+CHO ingestion on myomiR expression and their association with muscle protein synthesis (MPS). Twenty five adults performed 90 min of metabolically-matched (2.2 VO_2_ L·m^−1^) load carriage (LC; performed on a treadmill wearing a vest equal to 30% of individual body mass) or cycle ergometry (CE) exercise, during which EAA+CHO (10 g EAA and 46 g CHO) or non-nutritive control (CON) drinks were consumed. Expression of myomiR (RT-qPCR) were determined at rest (PRE), immediately post-exercise (POST), and 3 h into recovery (REC). Muscle protein synthesis (^2^H_5_-phenylalanine) was measured during exercise and recovery. Relative to PRE, POST, and REC expression of miR-1-3p, miR-206, miR-208a-5, and miR-499 was lower (*P* < 0.05) for LC compared to CE, regardless of dietary treatment. Independent of exercise mode, miR-1-3p and miR-208a-5p expression were lower (*P* < 0.05) after ingesting EAA+CHO compared to CON. Expression of miR-206 was highest for CE-CON than any other treatment (exercise-by-drink, *P* < 0.05). Common targets of differing myomiR were identified as markers within mTORC1 signaling, and miR-206 and miR-499 were inversely associated with MPS rates immediately post-exercise. These findings suggest the alterations in myomiR expression between exercise mode and EAA+CHO intake may in part be due to differing MPS modulation immediately post-exercise.

## Introduction

Exercise, essential amino acids (EAA), and insulin modulate anabolic signaling networks that regulate skeletal muscle protein synthesis (MPS) (Margolis and Rivas, 2015). These anabolic stimuli converge on the mechanistic target of rapamycin complex 1 (mTORC1), which when activated increases MPS (Drummond et al., 2009a; Pasiakos, 2012; Laplante and Sabatini, 2013). MPS and associated mTORC1 signaling responses to exercise are dependent on exercise type, as the mechanical strain of resistance-type exercise generally stimulates a greater increase in MPS compared to conventional endurance-type exercise (Wilkinson et al., 2008; Pasiakos et al., 2015). Consuming EAA during or after exercise potentiates the MPS response to exercise regardless of type (Tipton et al., 2004; Dreyer et al., 2008; Pasiakos et al., 2011; Dickinson et al., 2014; Rowlands et al., 2015). Co-ingesting carbohydrate with EAA during or after exercise optimizes the net anabolic response by eliciting an insulinemic response that exceeds the response to EAA alone (Tipton et al., 2001; Dreyer et al., 2008).

It has been suggested that acute alterations in skeletal muscle microRNA (myomiR; miR-1, miR-133a, miR-133b, miR-206, miR-208, and miR-499) expression may be sensitive to changes in the rate of MPS (Kirby and Mccarthy, 2013). myomiR expression has been shown to be acutely altered after exercise (Nielsen et al., 2010; Russell et al., 2013; Rivas et al., 2014) and EAA intake (Drummond et al., 2008, 2009b; Camera et al., 2016). The directionality of these changes appear to be sensitive to exercise type, and while the physiological function of such changes is not well described, altered myomiR expression may govern long-term muscle growth (Kirby and Mccarthy, 2013). For example, combining the contractile forces of resistance-type exercise with EAA ingestion downregulates miR-1 expression in young men (Drummond et al., 2008). These acute findings support long-term data by Mccarthy and Esser (2007) showing that, in a functional overload model (rodents), miR-1 and miR-133a expression is diminished during long-term periods of muscle hypertrophy. It is possible that downregulations in miR-1 and miR-133a expression facilitate increased anabolic signaling of the insulin-like growth factor-1(IFG-1)/insulin/mTORC1 pathway (Elia et al., 2009). Conversely, following more metabolically demanding, endurance-type exercise, which is generally not considered anabolic due to lower contractile forces than resistance-type exercise, miR-1, miR-133a, and miR-133b expression are upregulated when exercise is performed without EAA ingestion (Nielsen et al., 2010; Russell et al., 2013). The divergent myomiR responses to exercise mode and EAA intake may in part be triggered by altered MPS rates, however no study has directly tested this theory.

A recent investigation by our group reported that weighted endurance exercise stimulated a greater MPS response compared to conventional endurance exercise, and that EAA+CHO ingestion during exercise increased MPS to a greater extent than exercise alone (Pasiakos et al., 2015). Given these differences in MPS, muscle samples obtained from this previous study were used to determine if myomiR expression was diminished under conditions where MPS was enhanced. We hypothesized that the greater anabolic stimulus of weighted endurance exercise would be associated with reduced myomiR expression after exercise compared to conventional endurance exercise. Additionally, we anticipated that consuming EAA+CHO during exercise would suppress myomiR expression regardless of exercise mode.

## Methods

### Volunteers and experimental design

Twenty-five (23 males and 2 females) participated in this randomized, double-blind, placebo-controlled study after providing informed, written consent from October 2012 to November 2013 (Pasiakos et al., 2015). All study procedures were conducted at the US Army Research Institute of Environmental Medicine (USARIEM, Natick, MA). Volunteers were military personnel from the US Army Natick Research, Development and Engineering Center, Human Research Volunteer recruit platoon, and civilians from the local area. Volunteers were required to be between the ages of 18 and 39 years, weight stable (± 2 kg for a period of 2 months), physically fit (peak oxygen uptake, VO_2peak_ 40–60 mL·kg^−1^·min^−1^), and have a body mass index (BMI) between 22 and 29 kg·m^−2^. A medical screening was also conducted to ensure that potential volunteers could safely participate in the study. This study was approved by the Institutional Review Board at USARIEM and registered at www.clinicaltrials.gov as NCT01714479.

Volunteers were randomly assigned to one of four experimental groups. All four groups performed a single 90-min exercise bout. Two groups performed non-weight-bearing, conventional endurance exercise (cycle ergometry, CE), and the two remaining performed weighted endurance-type exercise (load carriage; LC). One of each of the exercise groups received combined EAA+CHO drinks to consume during exercise, and the other groups received flavor-matched, non-nutritive control (CON) drinks (LC-EAA+CHO: *n* = 6, CE-EAA+CHO: *n* = 7, LC-CON: *n* = 5, CE-CON: *n* = 7; Table 1). Muscle biopsies of the vastus lateralis were performed at rest (PRE), immediately post-exercise (POST), and after 3 h of recovery (REC), and analyzed for myomiR expression and mTORC1 signaling, and their associations with mixed-muscle protein synthesis (Pasiakos et al., 2015). Dietary intake and physical activity were carefully controlled to minimize any potential confounding effects on outcome variables as previously described (Pasiakos et al., 2015). Additionally, assessment of metabolic products (i.e., glucose, insulin, and amino acids) from blood assessing impact of endurance exercise mode and dietary supplementation has been previously published (Pasiakos et al., 2015).

**Table 1:** **Volunteer characteristics^a^**.

	**EAA+CHO**		**CON**	
	**Load carriage**	**Cycle ergometry**	**Load carriage**	**Cycle ergometry**
Age, y	23 ± 4	21 ± 2	24 ± 6	21 ± 2
Height, cm	177 ± 6	178 ± 8	178 ± 9	177 ± 8
Body mass, kg	80 ± 10	85 ± 12	80 ± 13	81 ± 10
Peak VO2, mL·kg^−1^·min^−1^	51 ± 4	49 ± 4	49 ± 5	50 ± 3

a *Data are means ± SD. LC-EAA+CHO (n = 6; load carriage + essential amino acid and carbohydrate supplement), CE-EAA+CHO (n = 7; cycle ergometry+ essential amino acid and carbohydrate supplement), LC-CON (n = 5; load carriage + non-nutritive control), and CE-CON (n = 7; cycle ergometry + non-nutritive control)*.

### Experimental load carriage and cycle ergometry

Volunteers performed LC by walking on a treadmill while wearing a weighted vest equivalent to 30% of baseline body mass. A Lode (BV, Netherlands) ergometer was used for the CE exercise bouts. Baseline VO_2*peak*_ and associated heart rates at maximal and submaximal levels were used to establish a target exercise intensity of 2.4 L·m^−1^ for both the LC and CE trials. Absolute kilocalorie expenditure was matched between modes by adjusting the speed and grade for LC and power for CE. By matching the energy cost, the effects of possible differences in mechanical force and contractile properties of LC and CE from the relative intensity and energy cost of the exercise bout were isolated. A 90-min familiarization trial was conducted at least 1 week before the experimental session to ensure the accuracy of the exercise prescription using indirect calorimetry (ParvoMedics, Sandy, UT).

The experimental LC and CE sessions were conducted in the morning following a 12-h fast. Volunteers began the 90-min intensity-matched LC or CE exercise bout after a muscle biopsy was taken from the vastus lateralis using aseptic technique (Pasiakos et al., 2015). Exercise intensity was verified using indirect calorimetry (and adjusted accordingly) every 30-min. The exercise intensity was not different between groups: oxygen uptake was 2.2 ± 0.1 L·m^−1^, energy expenditure was 1,000 ± 57 kcal·90-min^−1^, and average load carried for LC groups was 24 ± 3 kg. Volunteers consumed equal volumes (500 mL total, 125 mL per serving) of either the EAA+CHO (10 g EAA and 46 g carbohydrate, 223 kcal) or flavor-matched, non-nutritive CON (22 kcal, 5 g carbohydrate) drinks in 30-min intervals, beginning at the start of the exercise session and ending after completing the 90-min bout (Pasiakos et al., 2015). Additional muscle biopsies were taken from the same incision at POST and REC. Mixed-muscle protein synthesis (pre-cursor product model) during exercise and in recovery has been reported (Pasiakos et al., 2015) and presented in this paper to explore potential associations to expression of myomiR.

### myomiR expression

Total RNA was isolated in approximately 20 mg of muscle samples using miRNeasy Mini kit (Qiagen, Valencia, CA, USA). Quantity and quality of RNA were assessed using a Nandrop ND-1000 spectrophotometer (Nanodrop, Wilmington, DE, USA). Equal amounts of RNA were reverse transcribed using a TaqMan® microRNA RT kit (Applied Biosystems, Foster City, CA, USA). Real-time PCR using individual TaqMan® microRNA Assays (Applied Biosystems; miR-1-3p, miR-206, miR-208-5p, miR-133a, miR-133b, miR-499) was performed to assess myomiR expression. All myomiR were normalized to the geometric mean of RNU48 and 18S. Fold changes for myomiR were calculated using the ΔΔ cycle threshold (ΔΔC_*T*_) method (Pfaffl, 2001) and expressed relative to PRE.

### Prediction of myomiR target pathways

myomiR (miR-1-3p, miR-206, miR-208-5p, and miR-499) with altered expression in response to exercise mode or EAA+CHO were uploaded to miRWalk 2.0 (Dweep et al., 2011). This program allows for determination of miRNA targets through integration of several prediction software programs (miRWalk, RNA22, miRanda, and Targetscan). Kyoto Encyclopedia of Genes and Genomes (KEGG) pathway analysis was conducted to determine common anabolic signaling markers of relevant myomiR.

### Anabolic signaling

Approximately 30 mg of sample was homogenized in ice-cold RIPA (ThermoFisher, Waltham, MA, USA) homogenization buffer (1:10 wt/vol) containing 1 mM DTT, phosphatase (PhosSTOP™, Roche, Indianapolis, IN, USA), and protease (Complete™ ULTA Tablet, Roche) inhibitors. Homogenates were centrifuged for 15 min at 10,000 × g at 4°C, the supernatant (lysate) was collected, and protein content analyzed (ThermoFisher). Muscle lysates were solubilized in Laemmli buffer with equal amounts of total protein (15 μg) separated by SDS-PAGE using precast 4–20% Mini-PROTEAN TGX gels (Bio-Rad Laboratories, Hercules, CA, USA). Proteins were transferred to polyvinylidene fluoride membranes and exposed to commercially available primary antibodies specific to p-IRS^Ser302^, p-Akt^Ser473^, p-p70S6K^Thr389^, rpS6, p-rpS6^Ser235/236^ (Cell Signaling Technology, Danvers, MA, USA) at 4°C overnight. Labeling was performed using secondary antibody (anti-rabbit IgG conjugate with horseradish peroxidase; Cell Signaling Technology), and chemiluminescent reagent was applied (Super Signal, West Pico Kit; Pierce Biotechnology, Rockford, IL, USA). Blots were quantified using a phosphoimager (ChemiDoc XRS; Bio-Rad) and Image Lab software (Bio-Rad). Glyceraldehyde 3-phosphate dehydrogenase (GAPDH) was used to confirm equal protein loading per well. All data are presented as fold change relative to PRE.

### Statistical analyses

Mixed model repeated measures ANOVA was used to determine main effects and interactions of exercise mode (LC vs. CE), treatment (EAA+CHO vs. CON), and time (PRE, POST, and REC) for myomiR expression. Mixed model repeated measures ANOVA was also used to determine main effects and interactions for Western blot analysis. However, due to limited sample availability, Western blot analysis was only performed at PRE and POST. Bonferroni adjustments were used for *post hoc* comparisons if interactions were observed. The associations between myomiR expression and mixed-muscle protein synthesis were assessed using Spearman's Rho correlation coefficients. Significance was *P* < 0.05 and data were analyzed using SPSS (Version 21.0, 2010, SPSS Inc, Chicago, IL) and expressed as means ± SD.

## Results

### myomiR expression

**Figure 1:**
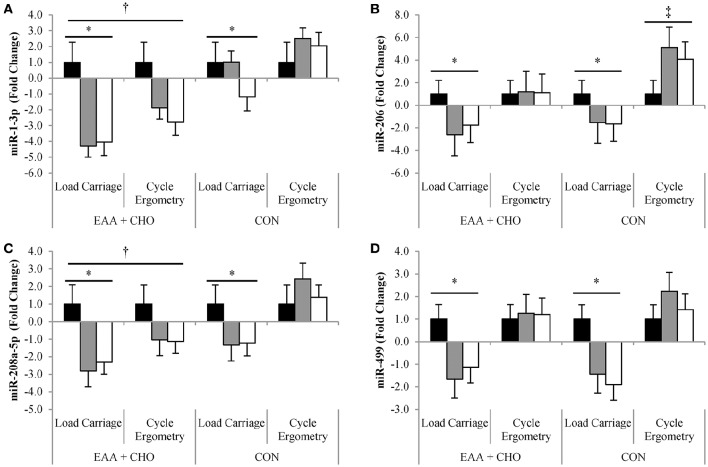
**Data are mean ± SD**. Expression of miR-1-3p **(A)**, miR-206 **(B)**, miR-208a-5p **(C)**, and miR-499 **(D)** at Baseline (■), Post-exercise (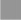) and Recovery (□). ^*^Load carriage different than Cycle Ergometry; *P* < 0.05. ^†^EAA+CHO (essential amino acid + carbohydrate) different than CON (non-nutritive control); *P* < 0.05. ^‡^Exercise-by-drink interaction; Cycle Ergometry-CON different than Cycle Ergometry-EAA+CHO; *P* < 0.05.

Expressions of miR-1-3p (*P* = 0.02), miR-206 (*P* < 0.01), miR-208a-5 (*P* = 0.01) and miR-499 (*P* < 0.01) were lower for LC compared to CE, regardless of dietary treatment (Figures 1A–D). miR-1-3p (*P* < 0.01) and miR-208a-5p (*P* = 0.03) expressions were lower after ingesting EAA+CHO compared to CON, independent of exercise mode. Expression of miR-206 was highest for CE+CON compared to any other treatment (exercise-by-drink, *P* = 0.001). There was no effect of exercise mode or dietary treatment on the expression of miR-133a-3p or miR-133b.

### Anabolic signaling

**Figure 2:**
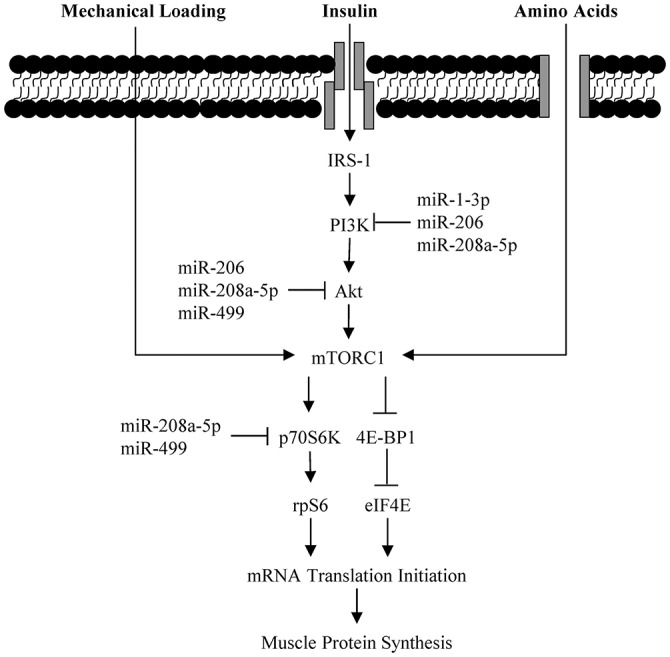
**Schematic of myomiR interaction with Akt-mTORC1 signaling pathway**.

**Figure 3:**
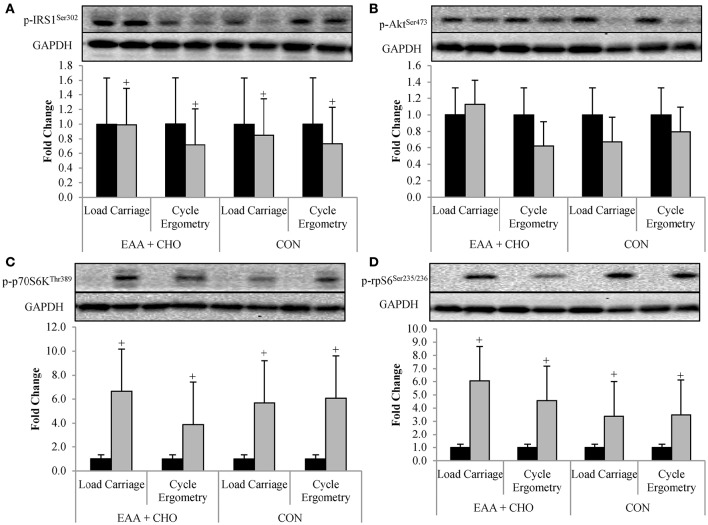
**Data are mean ± SD**. Phosphorylation status of IRS1^Ser302^ **(A)**, Akt^Ser473^ **(B)**, p70S6K^Thr389^ **(C)**, and rpS6^Ser235/236^ **(D)** at Baseline (■) and Post-exercise (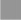).^+^Post-exercise different than Baseline; *P* < 0.05.

Bioinformatics analysis identified common targets of miR-1-3p, miR-206, miR-208a-5, and miR-499 were associated with insulin and mTORC1 signaling (Figure 2). Western blotting was used to assess the activation of these connected pathways. Phosphorylation of IRS^Ser302^ was 18 ± 36% lower (*P* = 0.046) POST compared to PRE, regardless of exercise mode and dietary treatment (Figure 3A). No effect of time, exercise mode, or dietary treatment was observed for phosphorylation of AKT^Ser473^ (Figure 3B). Independent of exercise mode and dietary treatment, phosphorylation of p70S6K^Thr389^ and rpS6^*Ser*235/236^ was 337 ± 262% and 457 ± 357%, respectively, higher at POST compared to PRE (Figures 3C, D, *P* < 0.01).

### Correlation of myomiR to muscle protein synthesis

**Figure 4:**
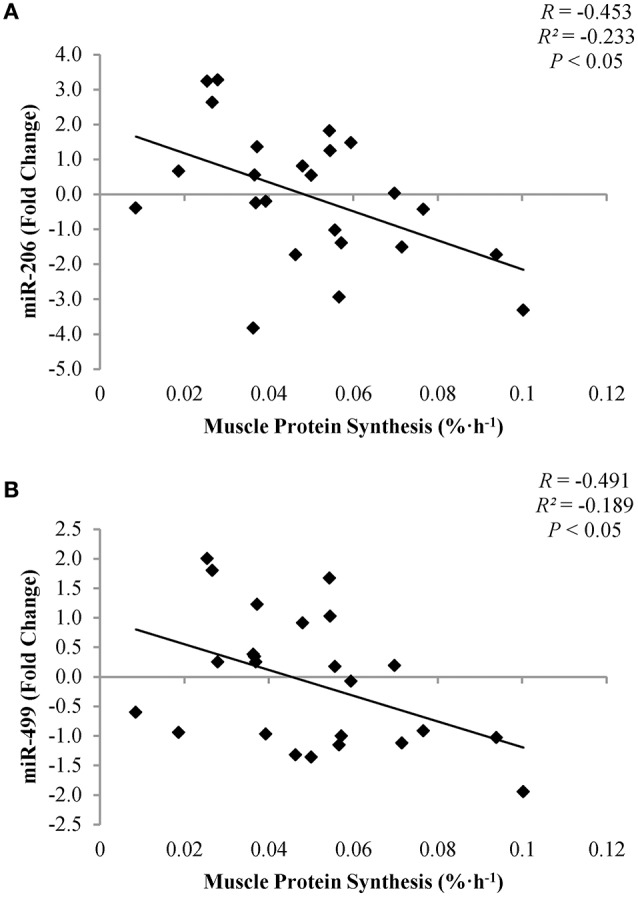
**Correlation of miR-206 (A)** and miR-499 **(B)** to muscle protein synthesis post-exercise.

Expression of miR-206 (*R* = −0.453; *P* = 0.03) and miR-499 (*R* = −0.491; *P* = 0.02) were negatively associated with MPS during exercise (Figures 4A, B). However, there was no correlation between expression of miR-206 (*R* = −0.173; *P* = 0.42) and miR-499 (*R* = −0.119; *P* < 0.58) to MPS during recovery. Expression of miR-1-3p or miR-208-5p was not correlated with muscle protein synthesis at any time point.

## Discussion

The primary outcome of this study was that myomiR expression differed by endurance exercise mode, independent of EAA+CHO supplementation. Weighted endurance-type exercise (LC) diminished miR-1-3p, miR-206, miR-208a-5p, and miR-499 expression, while conventional cycle ergometry endurance exercise (CE) increased myomiR expression. However, when CE was combined with ingestion of EAA+CHO, myomiR expression was either downregulated or remained the same immediately post and during the recovery phase, compared to baseline values. Furthermore, miR-206 and miR-499 expression was inversely associated with MPS during exercise, with higher rates of MPS indicative of lower myomiR expression. These findings suggest that alterations in the rate of MPS in response to acute anabolic stimulation by weighted endurance exercise and EAA+CHO intake may reduce myomiR expression.

The more anabolic weight-bearing endurance exercise resulted in diminished myomiR expression in the current study. In agreement with this findings, Mccarthy and Esser (2007) demonstrated that during periods of elevated muscle anabolism using a functional overload rodent model, miR-1 expression was reduced. Reduced miR-1 likely alters muscle anabolism by targeting upstream regulators of mTORC1. In differentiated C2C12 skeletal muscle cell culture experiments, overexpression of miR-1 reduces protein content (i.e., translation) of IGF-1 and its receptor (IGF-1R) (Elia et al., 2009). Binding of IGF-1 to its receptor phosphorylates IRS which stimulates Akt-dependent activation of mTORC1 (Schiaffino and Mammucari, 2011). With miR-1 induced reductions of IGF-1 and IGF-1R, phosphorylation of Akt^Ser473^ and total Akt protein content are also diminished, resulting in inhibition of cellular anabolism (Elia et al., 2009). Together these data suggest that during periods of anabolism there is a downregulation in myomiR expression.

While myomiR expression is reduced with anabolism, several investigations (Safdar et al., 2009; Nielsen et al., 2010; Russell et al., 2013) have reported that a bout of endurance exercise without EAA increases myomiR expression. Similarly, in the present investigation we report that CE without nutrient intervention resulted in an upregulation of myomiR expression post-exercise. Conventional endurance exercise is catabolic in nature, requiring increased mobilization of endogenous energy stores to support higher metabolic demands in skeletal muscle during exercise (Canto and Auwerx, 2009). Variant responses in myomiR expression in the present and past studies suggest that physiological state (i.e., anabolic or catabolic) of muscle likely dictates the direction of myomiR expression, and potentially function.

Consumption of EAA+CHO during exercise downregulated or attenuated the increase in myomiR expression immediately post-exercise and during recovery, regardless of exercise mode. Contrary to these findings, Drummond et al. (2009b) reported that 3 h after consumption of 10 g EAA at rest, miR-1, and miR-499 expression was upregulated. In the present study, participants consumed 10 g EAA with 46 g carbohydrate during a bout of weighted and conventional endurance exercise. The addition of mechanical strain from exercise may explain the differing results between our study and the findings by Drummond et al. (2009b). Supporting this statement, in an earlier investigation by Drummond et al. (2008), it was reported that miR-1 expression was reduced in young males when 20 g EAA were consumed immediately after a bout of resistance exercise. Together these findings suggest the mechanical strain of exercise may be required to amplify the greater anabolic stimulus and the downregulation of myomiR expression.

Despite no effect of exercise mode or EAA+CHO intake on mTORC1 signaling, myomiR expression was inversely associated with MPS immediately post-exercise. Results from the parent study (Pasiakos et al., 2015) showed that LC stimulated higher MPS rates compared to CE, independent of EAA+CHO. Furthermore, regardless of exercise mode, EAA+CHO elevated MPS to a greater extent than CON (Pasiakos et al., 2015). Divergent myomiR expression responses between exercise mode and EAA+CHO intake in the present analysis may in part be triggered by modulations in MPS. It has been hypothesized that alterations in myomiR expression after a bout of resistance exercise may be essential for underlying molecular adaptions resulting in muscle hypertrophy with chronic training (Kirby and Mccarthy, 2013). Concomitant reductions in myomiR expression with increased MPS rates post-exercise suggest a potential feed-forward mechanism, where acute anabolic stimulus (e.g., exercise and EAA) remove the negative inhibition of myomiR to initiate training adaptions through enhanced translation of mTORC1 associated proteins (Kong et al., 2008). Future time course analyses will be necessary to fully illustrate the potential mechanistic role of myomiR on anabolic training adaptions. Nevertheless, the present study provides supportive evidence of association between acute alterations myomiR expression and MPS immediately post-endurance exercise.

Though results from the present study are novel, there are limitations that must be considered. The inability to fully analyze recovery for possible concurrent alterations in myomiR expression with mTORC1 signaling limits the interpretation of our results. Phosphorylation status immediately post-exercise was highest for IRS1^Ser302^, Akt^Ser473^, p70S6K^Thr389^, and rpS6^Ser235/236^ after LC-EAA+CHO, yet no statistical differences were observed between exercise mode or EAA+CHO intake. The inability to determine statistical differences may be due to small sample size. Additionally, the lack of agreement between Western blot and myomiR results could be attributed in part to the time points mTORC1 signaling was assessed. While resistance and endurance exercise similarly upregulate p70S6K^Thr389^ and rpS6^Ser235/236^ immediately post-exercise (Wilkinson et al., 2008), phosphorylation of p70S6K^Thr389^ and rpS6^Ser235/236^ return to baseline values 4 h post-exercise after endurance exercise, but remain elevated after resistance exercise (Wilkinson et al., 2008). Therefore, if we had sufficient muscle sample to assess mTORC1 signaling in recovery, differences between endurance exercise mode and dietary treatment might have been magnified. The lack of muscle fiber typing by histology is another limitation of this investigation. While miR-1 and miR-133a appear to be uniformly expressed in all muscle fiber types, miR-206, miR-208, and miR-499 are more highly expressed in type I compared to type II muscle fibers (Mccarthy and Esser, 2007; Van Rooij et al., 2009; Kirby and Mccarthy, 2013). While we assessed myomiR expression relative to individual's baseline data to account for variance between participants, the magnitude of change in myomiR expression in response to exercise mode and EAA+CHO may be influenced by muscle fiber type.

In conclusion, these data show that myomiR expression is differentially regulated by exercise mode and EAA+CHO intake. After weight-bearing endurance exercise myomiR expression was lower compared to non-weight-bearing endurance exercise resulted in an upregulation of myomiR expression. Consuming EAA+CHO attenuated the increase in myomiR expression with CE, yet this suppression was more pronounced when EAA+CHO were consumed during LC. Common targets of differing myomiR were identified to be associated with mTORC1 signaling, and were inversely associated with MPS rate. Together these findings suggest alterations in myomiR expression between exercise mode and EAA+CHO intake may in part be due to acute modulation in MPS immediately post-exercise. With repeated exposure to combined weight-bearing exercise with essential amino acid and carbohydrate supplementation, diminished myomiR expression may facilitate muscle anabolic training adaptations to exercise by lowering mTORC1 inhibition.

### Grants

This work was supported by the U.S. Army Medical Research and Material Command and the US Army Natick Soldier Research Development and Engineering Center.

## Disclosures

The investigators adhered to the policies for protection of human subjects as prescribed in Army Regulation 70-25, and the research was conducted in adherence with the provisions of 32 CFR part 219. The opinions or assertions contained herein are the private views of the authors and are not to be construed as official or as reflecting the views of the Army or the Department of Defense. Any citations of commercial organizations and trade names in this report do not constitute an official Department of the Army endorsement of approval of the products or services of these organizations.

## Ethics statement

This study was carried out in accordance with the recommendations of the Institutional Review Board at USARIEM with written informed consent from all subjects. All subjects gave written informed consent in accordance with the Declaration of Helsinki. The protocol was approved by the Institutional Review Board at USARIEM.

## Author contributions

LM, HM, and SP conception and design of research; LM, HM, NM, CC, and SP performed experiments; LM, NM, CC, and SP analyzed data, interpreted results of experiments, prepared figures, and drafted manuscript; LM, HM, NM, CC, and SP revised the manuscript and approved final version.

## Funding

This work was supported by the US Army Medical Research and Material Command and the US Army Natick Soldier Research Development and Engineering Center.

### Conflict of interest statement

The authors declare that the research was conducted in the absence of any commercial or financial relationships that could be construed as a potential conflict of interest.

## References

[B1] Camera D. M. Ong J. N. Coffey V. G. Hawley J. A. (2016). Selective modulation of microrna expression with protein ingestion following concurrent resistance and endurance exercise in human skeletal muscle. Front. Physiol. 7:87. 10.3389/fphys.2016.00087 27014087 PMC4779983

[B2] Canto C. Auwerx J. (2009). PGC-1α, SIRT1 and AMPK, an energy sensing network that controls energy expenditure. Curr. Opin. Lipidol. 20, 98–105. 10.1097/MOL.0b013e328328d0a4 19276888 PMC3627054

[B3] Dickinson J. M. Gundermann D. M. Walker D. K. Reidy P. T. Borack M. S. Drummond M. J.. (2014). Leucine-enriched amino acid ingestion after resistance exercise prolongs myofibrillar protein synthesis and amino acid transporter expression in older men. J. Nutr. 144, 1694–1702. 10.3945/jn.114.198671 25332468 PMC4195415

[B4] Dreyer H. C. Drummond M. J. Pennings B. Fujita S. Glynn E. L. Chinkes D. L.. (2008). Leucine-enriched essential amino acid and carbohydrate ingestion following resistance exercise enhances mTOR signaling and protein synthesis in human muscle. Am. J. Physiol. Endocrinol. Metab. 294, E392–E400. 10.1152/ajpendo.00582.2007 18056791 PMC2706121

[B5] Drummond M. J. Dreyer H. C. Fry C. S. Glynn E. L. Rasmussen B. B. (2009a). Nutritional and contractile regulation of human skeletal muscle protein synthesis and mTORC1 signaling. J. Appl. Physiol. (1985) 106, 1374–1384. 10.1152/japplphysiol.91397.2008 19150856 PMC2698645

[B6] Drummond M. J. Glynn E. L. Fry C. S. Dhanani S. Volpi E. Rasmussen B. B. (2009b). Essential amino acids increase microRNA-499, -208b, and -23a and downregulate myostatin and myocyte enhancer factor 2C mRNA expression in human skeletal muscle. J. Nutr. 139, 2279–2284. 10.3945/jn.109.112797 19828686 PMC2777476

[B7] Drummond M. J. Mccarthy J. J. Fry C. S. Esser K. A. Rasmussen B. B. (2008). Aging differentially affects human skeletal muscle microRNA expression at rest and after an anabolic stimulus of resistance exercise and essential amino acids. Am. J. Physiol. Endocrinol. Metab. 295, E1333–E1340. 10.1152/ajpendo.90562.2008 18827171 PMC2603551

[B8] Dweep H. Sticht C. Pandey P. Gretz N. (2011). miRWalk–database: prediction of possible miRNA binding sites by “walking” the genes of three genomes. J. Biomed. Inform. 44, 839–847. 10.1016/j.jbi.2011.05.002 21605702

[B9] Elia L. Contu R. Quintavalle M. Varrone F. Chimenti C. Russo M. A.. (2009). Reciprocal regulation of microRNA-1 and insulin-like growth factor-1 signal transduction cascade in cardiac and skeletal muscle in physiological and pathological conditions. Circulation 120, 2377–2385. 10.1161/CIRCULATIONAHA.109.879429 19933931 PMC2825656

[B10] Kirby T. J. Mccarthy J. J. (2013). MicroRNAs in skeletal muscle biology and exercise adaptation. Free Radic. Biol. Med. 64, 95–105. 10.1016/j.freeradbiomed.2013.07.004 23872025 PMC4867469

[B11] Kong Y. W. Cannell I. G. De Moor C. H. Hill K. Garside P. G. Hamilton T. L.. (2008). The mechanism of micro-RNA-mediated translation repression is determined by the promoter of the target gene. Proc. Natl. Acad. Sci. U.S.A. 105, 8866–8871. 10.1073/pnas.0800650105 18579786 PMC2449332

[B12] Laplante M. Sabatini D. M. (2013). Regulation of mTORC1 and its impact on gene expression at a glance. J. Cell Sci. 126, 1713–1719. 10.1242/jcs.125773 23641065 PMC3678406

[B13] Margolis L. M. Rivas D. A. (2015). Implications of exercise training and distribution of protein intake on molecular processes regulating skeletal muscle plasticity. Calcif. Tissue Int. 96, 211–221. 10.1007/s00223-014-9921-0 25348078 PMC6691734

[B14] Mccarthy J. J. Esser K. A. (2007). MicroRNA-1 and microRNA-133a expression are decreased during skeletal muscle hypertrophy. J. Appl. Physiol. (1985) 102, 306–313. 10.1152/japplphysiol.00932.2006 17008435

[B15] Nielsen S. Scheele C. Yfanti C. Akerstrom T. Nielsen A. R. Pedersen B. K.. (2010). Muscle specific microRNAs are regulated by endurance exercise in human skeletal muscle. J. Physiol. 588, 4029–4037. 10.1113/jphysiol.2010.189860 20724368 PMC3000590

[B16] Pasiakos S. M. (2012). Exercise and amino acid anabolic cell signaling and the regulation of skeletal muscle mass. Nutrients 4, 740–758. 10.3390/nu4070740 22852061 PMC3407992

[B17] Pasiakos S. M. Mcclung H. L. Margolis L. M. Murphy N. E. Lin G. G. Hydren J. R.. (2015). Human muscle protein synthetic responses during weight-bearing and non-weight-bearing exercise: a comparative study of exercise modes and recovery nutrition. PLoS ONE 10:e0140863. 10.1371/journal.pone.0140863 26474292 PMC4608805

[B18] Pasiakos S. M. Mcclung H. L. Mcclung J. P. Margolis L. M. Andersen N. E. Cloutier G. J.. (2011). Leucine-enriched essential amino acid supplementation during moderate steady state exercise enhances postexercise muscle protein synthesis. Am. J. Clin. Nutr. 94, 809–818. 10.3945/ajcn.111.017061 21775557

[B19] Pfaffl M. W. (2001). A new mathematical model for relative quantification in real-time RT-PCR. Nucleic Acids Res. 29:e45. 10.1093/nar/29.9.e45 11328886 PMC55695

[B20] Rivas D. A. Lessard S. J. Rice N. P. Lustgarten M. S. So K. Goodyear L. J.. (2014). Diminished skeletal muscle microRNA expression with aging is associated with attenuated muscle plasticity and inhibition of IGF-1 signaling. FASEB J. 28, 4133–4147. 10.1096/fj.14-254490 24928197 PMC5058318

[B21] Rowlands D. S. Nelson A. R. Phillips S. M. Faulkner J. A. Clarke J. Burd N. A.. (2015). Protein-leucine fed dose effects on muscle protein synthesis after endurance exercise. Med. Sci. Sports Exerc. 47, 547–555. 10.1249/MSS.0000000000000447 25026454

[B22] Russell A. P. Lamon S. Boon H. Wada S. Guller I. Brown E. L.. (2013). Regulation of miRNAs in human skeletal muscle following acute endurance exercise and short-term endurance training. J. Physiol. 591, 4637–4653. 10.1113/jphysiol.2013.255695 23798494 PMC3784204

[B23] Safdar A. Abadi A. Akhtar M. Hettinga B. P. Tarnopolsky M. A. (2009). miRNA in the regulation of skeletal muscle adaptation to acute endurance exercise in C57Bl/6J male mice. PLoS ONE 4:e5610. 10.1371/journal.pone.0005610 19440340 PMC2680038

[B24] Schiaffino S. Mammucari C. (2011). Regulation of skeletal muscle growth by the IGF1-Akt/PKB pathway: insights from genetic models. Skelet. Muscle 1:4. 10.1186/2044-5040-1-4 21798082 PMC3143906

[B25] Tipton K. D. Elliott T. A. Cree M. G. Wolf S. E. Sanford A. P. Wolfe R. R. (2004). Ingestion of casein and whey proteins result in muscle anabolism after resistance exercise. Med. Sci. Sports Exerc. 36, 2073–2081. 10.1249/01.MSS.0000147582.99810.C5 15570142

[B26] Tipton K. D. Rasmussen B. B. Miller S. L. Wolf S. E. Owens-Stovall S. K. Petrini B. E.. (2001). Timing of amino acid-carbohydrate ingestion alters anabolic response of muscle to resistance exercise. Am. J. Physiol. Endocrinol. Metab. 281, E197–E206. 11440894 10.1152/ajpendo.2001.281.2.E197

[B27] Van Rooij E. Quiat D. Johnson B. A. Sutherland L. B. Qi X. Richardson J. A.. (2009). A family of microRNAs encoded by myosin genes governs myosin expression and muscle performance. Dev. Cell 17, 662–673. 10.1016/j.devcel.2009.10.013 19922871 PMC2796371

[B28] Wilkinson S. B. Phillips S. M. Atherton P. J. Patel R. Yarasheski K. E. Tarnopolsky M. A.. (2008). Differential effects of resistance and endurance exercise in the fed state on signalling molecule phosphorylation and protein synthesis in human muscle. J. Physiol. 586, 3701–3717. 10.1113/jphysiol.2008.153916 18556367 PMC2538832

